# An Immunocompromised Man With a Chest Wall Mass

**DOI:** 10.1016/j.acepjo.2025.100225

**Published:** 2025-07-15

**Authors:** Patrick M. Lin, Julia Wickman, Jonathon Haverty

**Affiliations:** Department of Emergency Medicine, Hackensack University Medical Center, 30 Prospect Ave, Hackensack, NJ 07601, USA

## 1 Patient Presentation

**Figure 1:**
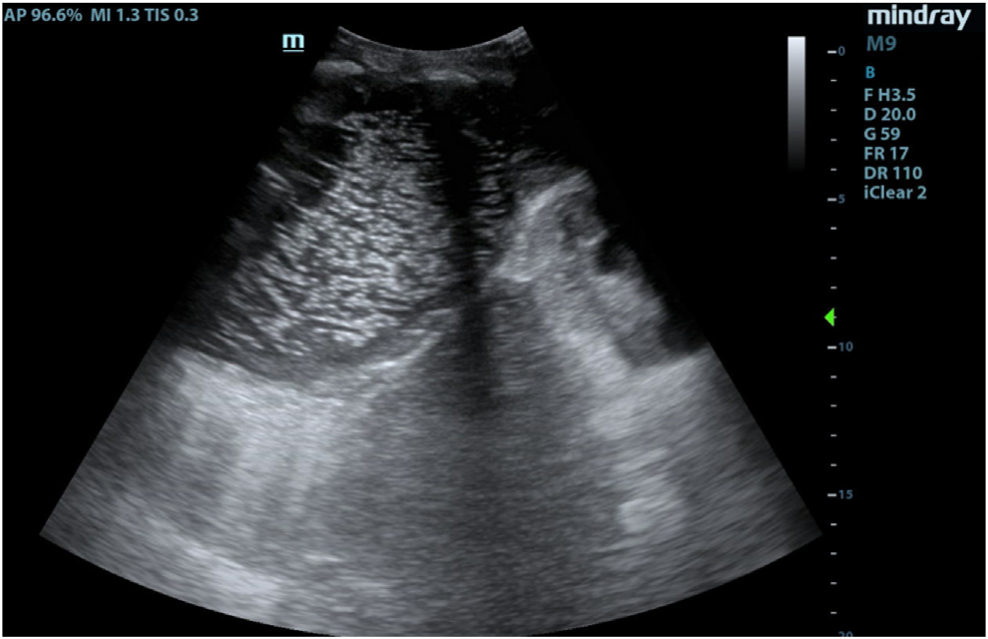
A large echogenic, punctiform pleural collection is noted cranial to the diaphragm, with the right kidney seen caudal to the diaphragm.

A 57-year-old male with a history of Crohn’s disease on infliximab presented to the emergency department with 1 month of dyspnea and a progressively expanding chest wall “mass.” Examination showed an erythematous, fluctuant area over the patient’s right lateral chest wall. His vitals were normal, other than tachycardia at 107 bpm. Subcutaneous incision and drainage yielded 400 mL of brisk, purulent drainage. Point-of-care ultrasound (POCUS) of the right posterolateral lung zone demonstrated a large echogenic punctiform collection in the pleural space, communicating with the skin. The collection demonstrated positive “spine sign,” and B-lines were visualized emanating from the posterior pleura (Fig 1, Video 1).

**Video 1:**
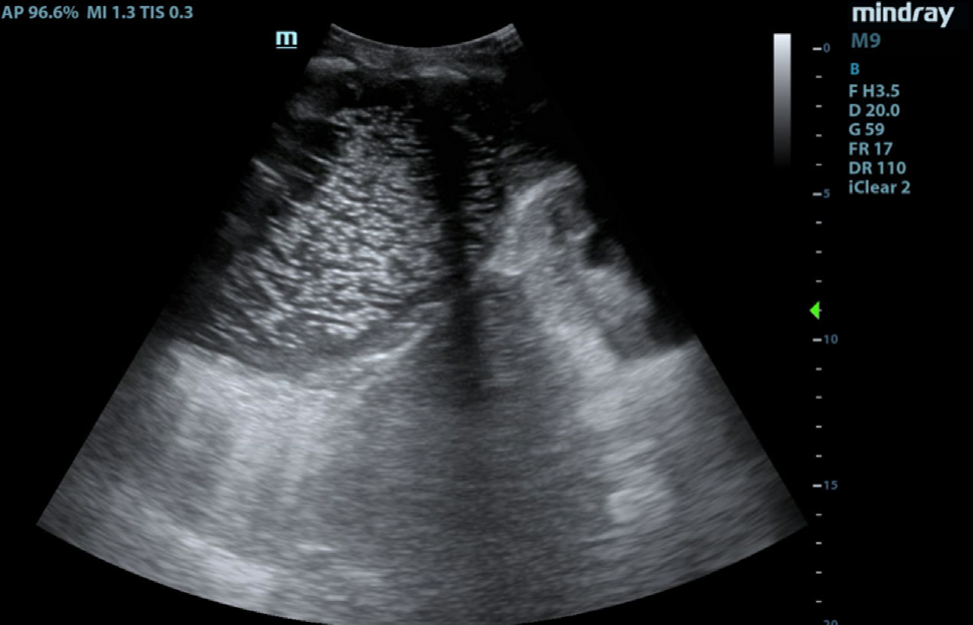
The spine sign is noted as a continuous hyperechoic line far-field to the pleural space. Confluent B-lines are seen emanating from the posterior pleura.

## 2 Diagnosis: Empyema Necessitans

**Figure 2:**
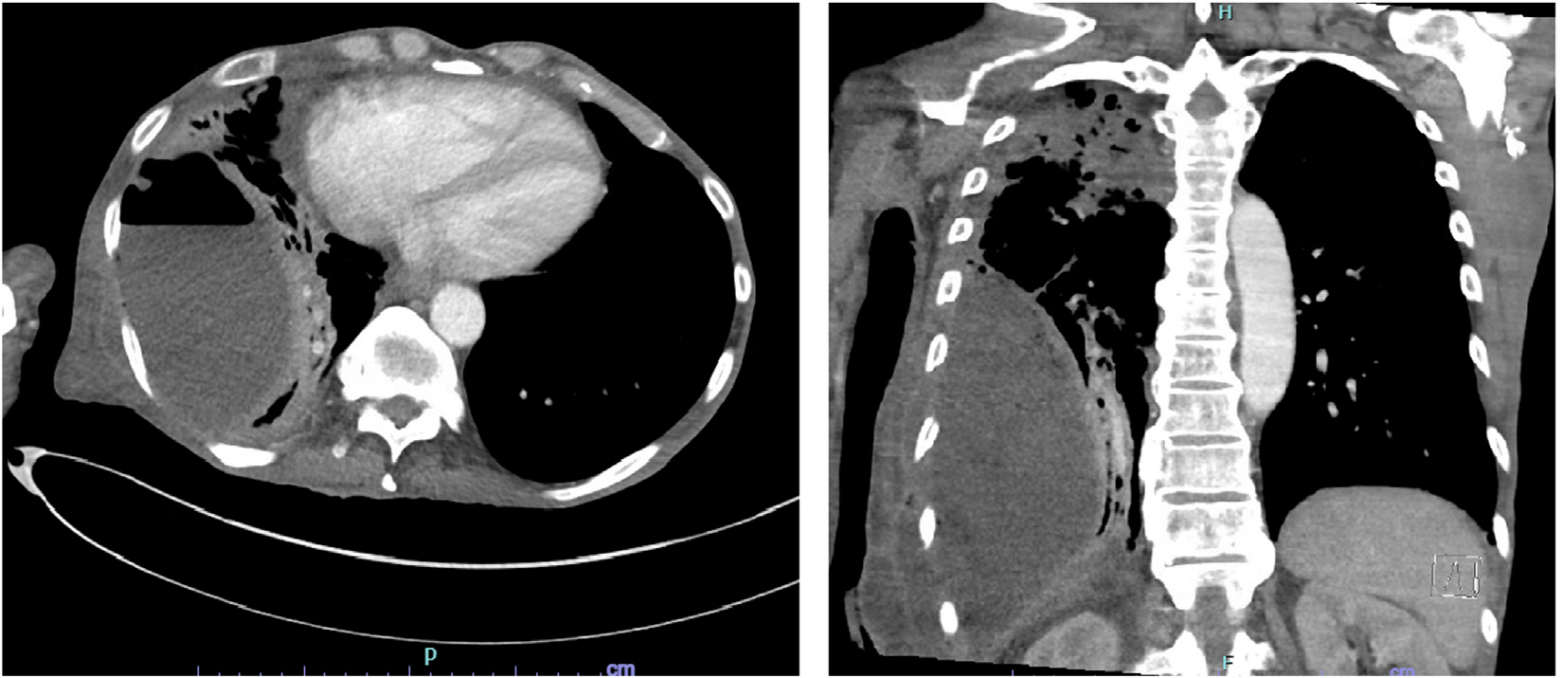
Computed tomography imaging demonstrated a 1-L collection lateral to the right lower lobe containing an air-fluid level. Multifocal consolidation and scarring to the right lung were noted.

Empyema necessitans (EN) describes a rare complication of empyema where the purulent infection dissects beyond the pleural space into neighboring chest wall musculature and subcutaneous tissue.^1^^,^^2^ Our case initially appeared to be a skin abscess, but ultrasonographic signs suggested an exudative pleural effusion, which was confirmed to be EN on computed tomography (Fig 2).^3^^,^^4^ The collection required extensive operations, including video-assisted thoracoscopic surgery, open thoracotomy, and lung decortication. The patient was discharged home ambulatory on postoperative day 7.

EN is typically found in immunocompromised and postoperative patients, although nowadays, it is rare due to the prevalence of antibiotics. Diagnosis of EN is often difficult due to its masquerading as a subcutaneous abscess. Thus, our report reinforces the use of POCUS as a quick, accurate tool for the early detection of EN. POCUS changed management, guiding further thoracic imaging and involvement of a multidisciplinary surgical team, ultimately leading to the resolution of the patient’s condition.

## Funding and Support

By *JACEP Open* policy, all authors are required to disclose any and all commercial, financial, and other relationships in any way related to the subject of this article as per ICMJE conflict of interest guidelines (see www.icmje.org). The authors have stated that no such relationships exist.
